# A Retrospective Chart Review of Ostomy Pouching Systems in New Ileostomy Patients: A Sub-Analysis

**DOI:** 10.3390/nursrep15060206

**Published:** 2025-06-06

**Authors:** Cecilia Zamarripa, Alexandra Craig, Carol Mathews, Lisa Small, Amy Folk

**Affiliations:** 1 Wound Ostomy Continence Department, University of Pittsburgh Medical Center Presbyterian, Pittsburgh, PA 15213, USA; craiga11@upmc.edu; 2 Wound Ostomy Continence Department, University of Pittsburgh Medical Center Shadyside, Pittsburgh, PA 15232, USA; waynecarol608@verizon.net (C.M.); folkal@upmc.edu (A.F.); 3 Coloplast Corp, 1601 West River Road North Minneapolis, Minneapolis, MN 55411, USA

**Keywords:** ostomy, stoma, ileostomy, colostomy, urostomy, post-operative, leakage, peristomal skin complications, peristomal skin conditions, ostomy barrier

## Abstract

**Background/Objectives:** Ostomy creation surgery is a common intervention for patients with conditions such as colorectal cancer, ulcerative colitis, Crohn’s disease, or acute events like trauma and gastrointestinal perforation. Individuals with an ileostomy face unique challenges when managing their new ostomies due to the liquid caustic nature of the effluent, increasing the likelihood of leakage and peristomal skin complications (PSCs). This sub-analysis evaluates the prevalence of leakage and PSCs in a cohort of individuals with a new ileostomy and examines the risk of leakage of different ostomy pouching systems and their impact on leakage and PSCs. **Methods:** This sub-analysis examined a cohort of 98 patients from a previously published retrospective chart review of stoma-creation surgeries at the University of Pittsburgh Medical Center. Data on pouching system selection, leakage, and PSCs were collected from electronic medical records and evaluated across 479 pouch changes. Two main barrier pouching systems were analyzed: elastic tapeless border (ETB) and ceramide-infused tape-border (CIB) barriers. Statistical analyses using generalized linear mixed models assessed the risk of leakage for each barrier type and controlled for significant differences in the sub-groups. **Results:** The prevalence of leakage in the ileostomy cohort was 19%, with the prevalence of leakage increasing over successive pouch changes. The ETB sub-group experienced a significantly lower risk of leakage (13.7%) compared to CIB (29.3%), reflecting a 53.2% lower risk of leakage with ETB (*p* = 0.03; OR 2.45). **Conclusions:** This sub-analysis of ileostomy patients confirms that ETB significantly reduces the risk of leakage in this more difficult to manage population compared to CIB, a clinically important consideration in PSC development and overall ostomy management. Evidence-based selection of ostomy barriers can improve patient outcomes, enhance quality of life, and reduce healthcare resource utilization.

## 1. Introduction

Ostomy creation surgery is a common intervention for individuals with chronic conditions such as colorectal cancer, ulcerative colitis (UC), Crohn’s disease (CD), or in response to acute events such as trauma or gastrointestinal perforation, which has been associated with diverticulitis [1]. Additionally, ostomy creation may be necessitated for the treatment and management of congenital anomalies, incontinence, and spinal cord injury [2]. Epidemiological studies estimate that between 100,000 and 150,000 new ostomy surgeries are performed annually in the United States, and of those, approximately 32.2% are ileostomies, with the most common indications for surgery being colorectal cancer and inflammatory bowel disease [3,4].

Postoperative care following new ostomy surgery involves the use of a barrier pouching system designed to contain stoma effluent effectively and maintain peristomal skin integrity. Optimal ostomy management relies on selecting a barrier pouching system which consistently provides a reliable and secure seal throughout its application duration (wear time), ensuring patient comfort and reducing the potential for complications [5]. In the United States, there are several ostomy pouching system brands and types of barriers available with different physical characteristics and attributes designed to collect effluent and protect the peristomal skin from leakage (e.g., elastic tapeless border (ETB) barrier vs. tape-border barrier; 1-piece system versus 2-piece system).

Rates of leakage reported in the scientific literature for individuals immediately following ostomy creation surgery vary widely; 18–76% of patients living with an ostomy experience leakage, and 37% of them experience leakage at least once per week [6,7]. The presence of leakage has been shown to increase the risk of peristomal skin complications (PSCs) and patient anxiety, putting them at higher risk for emergency room (ER) visits and hospital readmissions, resulting in preventable healthcare utilization and unnecessary ostomy pouching system consumption [6,7,8,9]. In a recent publication by Zamarripa et al., the prevalence of leakage among 214 patients who underwent ostomy surgery was 19% during their stay in the acute setting [7].

Ileostomy effluent is semi-liquid to liquid because the stoma output leaves the bowel before reaching the colon, preventing water and electrolyte absorption. In normal physiology, the colon absorbs fluids and neutralizes digestive enzymes, but in ileostomies, these enzymes have not yet been neutralized, resulting in the effluent being both liquid and corrosive on the skin, increasing the risk of PSCs if leakage occurs. While people with an ostomy of any type have a lifetime cumulative probability for developing PSCs at 43%, ileostomy patients are at a greater risk of PSCs, with a lifetime probability range from 36% to as high as 73% [9,10,11,12].

In some individuals with an ostomy, high volumes of output (>1000 mL/day) can occur, leading to serious electrolyte imbalances, dehydration, and difficulty managing leakage, resulting in unplanned provider appointments, emergency room visits, or hospital readmissions [13,14,15]. Among individuals with an ileostomy, the all-cause readmission rate is more than 50% within 120 days of ostomy surgery, and, specifically, in the first 30 days following discharge after ileostomy creation surgery, 20% of patients will be readmitted [4,13,16].

While we are not aware of any studies directly correlating rates of leakage and PSCs among different ostomy types, given the liquid and caustic nature of effluent for individuals with an ileostomy and high effluent volume output, the ostomy care of these individuals may need to be managed more rigorously to minimize leakage and PSCs. Subsequently, selecting well-fitting, secure barriers should result in predictable change frequency and no leakage, which is key to maintaining peristomal skin integrity and reducing leakage in this higher-risk population.

## 2. Materials and Methods

This sub-analysis focuses on patients with newly created ileostomies, drawn from the broader cohort of a previously published retrospective chart review on stoma creation surgeries at the University of Pittsburgh Medical Center (UPMC) [7]. This study was conducted at two affiliated UPMC hospitals (Presbyterian and Shadyside campuses) and reviewed medical charts of new ostomy patients admitted between February 2022 and June 2023. Charts that met the inclusion criteria were reviewed by WOC nurses, who extracted pre-defined data points for analysis. Data collection included up to five observations (defined as pouch changes) per patient, starting from the initial pouching system placed in the operating room through discharge or 14 days, whichever was longer. Based on the standard of care, leakage was defined as any effluent present under the baseplate, visible before or during barrier removal. PSCs were defined as any change to the peristomal skin area, including redness, blisters, irritant contact dermatitis, medical adhesive-related skin damage, and fungal dermatitis, and the diagnosis of a PSC was based on assessment by a WOC nurse. The study protocol was reviewed by the University of Pittsburgh Institutional Review Board (STUDY22080173) and deemed to be exempt from requiring informed consent.

For this sub-analysis, we examined demographic and clinical variables specific to ileostomy patients, as well as outcomes related to the performance of two predominant barrier types. The main outcomes of leakage and PSCs were selected due to clinical relevance. No new data were collected; all analyses were performed on the existing dataset. Three main brands (manufacturers) of pouching systems were used during the study period (Coloplast A/S (Humlebaek, Denmark), Hollister (Libertyville, IL, USA), and Convatec (Reading, UK)). Product selection, including brand, barrier type, and accessories, was based on WOC nurse clinical judgment and availability within the hospital formulary. Both hospital formularies included access to all three major manufacturers. As a retrospective study, all data reflect the standard of care during the study window.

### 2.1. Data Collection—Leakage and Peristomal Skin Complications (PSCs)

Leakage and PSCs were recorded as binary outcomes (present or absent) based on medical record documentation. If available, the degree of leakage and PSC severity were captured. Leakage was documented in the medical record by a WOC nurse or documented by other nursing staff when a WOC nurse was unavailable. Data were extracted directly from the selected charts into a standardized collection form, which contained dropdown options for most data points to reduce subjectivity.

### 2.2. Data Extraction and Analysis

An independent biostatistician performed all analyses using SPSS software, version 29 (SPSS, Chicago, IL, USA). Observation-level analyses (per pouch change by a WOC nurse) for binary outcomes were conducted using generalized linear mixed models (GENLINMIXED command) with the probability distribution set to binomial, the link function set to logit, and with patient ID as the subject variable. The predicted probability of leakage was calculated through the SAVE subcommand of the GENLINMIXED command. Predicted probabilities of leakage (risk of leakage) were generated and averaged for the two most common barrier brands—SenSura^®^ Mio, an elastic tapeless barrier (ETB, manufactured by Coloplast), and CeraPlus™, a ceramide-infused tape-border barrier (CIB, manufactured by Hollister).

The multivariate model compared the risk of leakage and controlled for relevant covariates: surgery indication (chronic versus acute), surgery type (elective versus emergent), surgery duration, stoma type (end versus loop), and chronic steroid use (>30 days).

The prevalence of leakage was reported as the actual (observed) occurrence of leakage across all observations (pouch changes). Descriptive statistics were used to characterize patient demographics, and endpoints for this sub-analysis were consistent with those analyzed in the original publication [7].

## 3. Results

In total, 98 out of 214 patients from the original, full study population underwent new ileostomy creation surgery. We will refer to these 98 patients as the ileostomy cohort.

### 3.1. Demographic and Surgical Characteristics in the Ileostomy Cohort and Full Study Population

The age of individuals with a new ileostomy ranged from 26 to 94 years old; the mean age was 59.6 years old. They were predominantly white (84.7%, n = 83), 53.1% were male (n = 51), and the average BMI was 27.9 kg/m^2^ (range 14.1–48.5 kg/m^2^). Chronic conditions were the most common reasons for surgery (54.1%; n = 53), and surgeries were primarily elective (76.5%, n = 75). Chronic conditions included diagnoses of cancer, inflammatory bowel disease (Crohn’s), diversion for wound management, and other chronic indications. Surgical indications for acute conditions included diagnoses of bowel perforation, diverticulitis, fecal diversion, ischemia, trauma, and other acute conditions.

Demographic and surgical characteristics of the ileostomy cohort were compared to those of the original, full study population, and several statistically significant differences were noted: mean age, surgical approach, mean duration of surgery, loop versus end stoma, rod use, and effluent type. Individuals with an ileostomy, on average, were younger (59.6 years versus 67.6 years; *p* < 0.001), with a longer duration of surgery (277 min versus 252 min; *p* = 0.049) compared to the full study population. Emergent surgeries occurred to a greater extent in the ileostomy cohort compared to the total study population (45.9% versus 38.8%), and an open procedure was used in 87.8% of ileostomy surgeries versus 82.2% in the full population. The resulting stomas were end stomas 51% of the time in ileostomies compared to 66.8% in the original, full study population (*p* < 0.001). Ileostomies were more commonly associated with rod placement compared to the original, full study subjects (45.9% versus 33.2% (*p* < 0.001)). The use of steroids and the average BMI were similar between the ileostomy cohort and the total study population, although a greater proportion of individuals with an ileostomy had a BMI > 30 (35.7% vs. 29.3%). All other parameters analyzed between the two groups (ileostomy vs. full study population) were considered similar, with no statistically significant differences. Table 1 summarizes the demographic and ostomy surgery characteristics of the two populations analyzed.

### 3.2. Length of Stay and Pouch Change Characteristics of the Ileostomy Cohort

The average length of stay (LOS) for individuals with a new ileostomy was 9.5 days (SD 4.1; range 1–14), with 65.3% hospitalized for less than two weeks (n = 64), and 85.7% had a first pouch change within 48 h of surgery. Comparatively, average LOS for the total study population was 7.3 days (SD 4; range 1–13) and average time to first pouching system change was 1.5 days (SD 1.0; range 0–5). All patients, both in the total study population and in the ileostomy cohort, had at least one pouch change prior to hospital discharge, and collectively, the ileostomy cohort had a total of 479 pouch changes compared to 832 in the total study population.

All individuals with ileostomies left the operating room in a flat pouching system, and the majority had a pouching system with an elastic tapeless border (ETB) barrier or ceramide-infused tape border (CIB) barrier [61.2%, n = 60; 38.8%, n = 38], respectively. Across the 479 pouch changes, the predominant barrier shape was flat (78.4%, n = 374), and more two-piece pouching systems were used than one-piece (64.3% (n = 308) versus 35.5% (n = 170)). The two most used brands of pouching systems were Coloplast and Hollister. Similarly, in the total study population, all individuals left the operating room in a flat pouching system, and over the entire study period, 71% (n = 587) were flat barriers and predominantly two-piece systems (65%; 537), with the most common brands also being Coloplast and Hollister.

### 3.3. Leakage in the Ileostomy Cohort and Full Study Population

**Figure 1:**
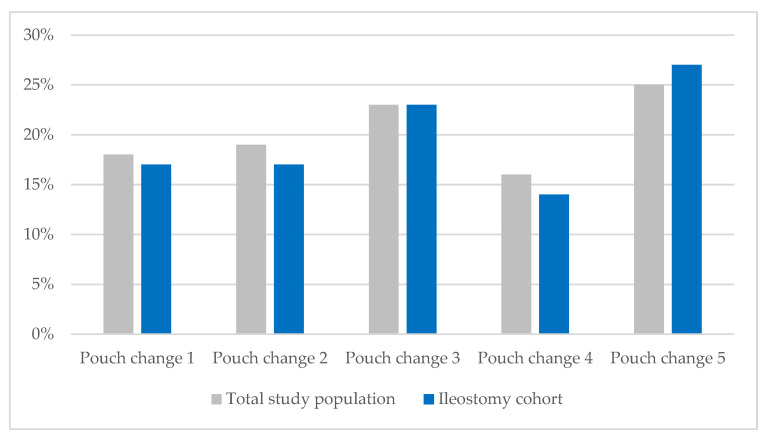
Prevalence of leakage by observation.

Every observation (pouch change) was evaluated by a WOC nurse to determine whether the previously placed barrier leaked. As previously reported for the full study population, the percentage of patients experiencing leakage rose from 17% after the first pouch change to 25% by the fifth. Likewise, the percentage of individuals with ileostomies experiencing leakage increased from 17% to 27% during their acute care stay (Figure 1). In the ileostomy cohort, liquid output was observed in 88.8% (n = 87) of patients at the first pouch change, and 80.6% (n = 79) experienced at least one leakage event during their acute care stay, with 19.4% (n = 19) of individuals with an ileostomy having two or more leakage events. The overall prevalence of leakage in the ileostomy cohort was 19%.

The ileostomy cohort was also analyzed across patient demographics and surgical characteristics of the ETB and CIB sub-groups for observations (pouch changes) one through five. The ETB and CIB barrier groups differed in terms of gender, average BMI, ethnicity, steroid use, mean age, surgical approach, loop versus end, and stoma description. These differences were controlled by the statistical methodology used. Risk of leakage was analyzed by barrier type (ETB and CIB) for all 368 pouch change observations in ileostomy patients after adjusting for the statistically significant variables; 255 (69.3%) observations of the ileostomy cohort involved the use of an ETB, while 113 (30.7%) observations noted the use of CIB (Table 2). The risk of leakage for CIB was 29.3% (95% CI: 26–33%), while ETB had a significantly lower risk of leakage at 13.7% (95% CI: 12–15%). ETB was associated with a 53.2% lower risk of leakage compared to CIB, a statistically significant difference (*p* = 0.03). The odds of leakage were 2.45 times higher with CIB than ETB (OR = 2.45; 95% CI: 1.08–5.60).

This statistically significant reduction in risk of leakage was consistent with findings in the total study population with 15% for ETB (95% CI: 14–16%) and 26% for CIB (95% CI: 24–28%) (*p* = 0.011). In the total study population, the odds of leakage were 1.95 times higher with CIB than ETB.

### 3.4. Peristomal Skin Complications

**Figure 2:**
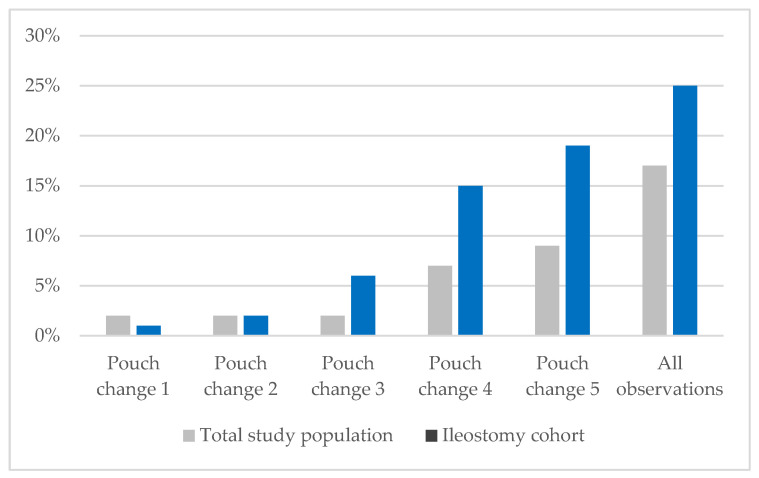
Prevalence of peristomal skin complications by observation.

The presence of PSCs was documented in 25% (n = 25) of the 98 patients with ileostomy, which is higher than the 17% (n = 36) prevalence of PSCs in the full study population (n = 214) [7]. In both analyses (original and ileostomy sub-analysis), the prevalence of PSCs increased as LOS increased (Figure 2). Consistent with the original, total study population, individuals with ileostomies exhibited five types of PSCs—irritant contact dermatitis, cutaneous blisters, medical adhesive-related skin damage, and fungal dermatitis. The prevalence of PSCs in ileostomies increased throughout the duration of the acute care stay and was highest at pouch change 5 (19%). Given the small number of PSCs at each observation (pouch change) for the ileostomy cohort, no statistical analysis was conducted comparing PSCs in the ETB and CIB sub-groups.

## 4. Discussion

Given the growing incidence of colorectal cancer in younger patients and the development of inflammatory bowel disease in increasingly westernized countries, it is not unexpected that the mean age for ileostomies in this sub-analysis was lower compared to the full study population in the original publication [7,16,17]. The differences in surgical approach and average duration of surgery are likely to be a result of the primary indication for surgery [18]. Individuals with an ileostomy have been regarded by clinicians as harder to manage because of predominantly liquid and caustic effluents and the higher volume of output, making it more difficult to find a secure, well-fitting barrier—albeit these elements are essential in ostomy management and PSC prevention. A key component of identifying a secure, well-fitting barrier is to proactively minimize the likelihood of leakage in ostomy patients. The ileostomy sub-group analysis demonstrated that ETB is associated with a statistically significant and clinically meaningful reduction in the risk of leakage compared to CIB. The mechanism underlying the difference in leakage risk between barrier types may be attributed to the ETB’s elastic adhesive technology, which enhances conformity to the peristomal skin, maintaining a secure seal during postoperative movement and healing. Further research should explore patient characteristics prone to leakage and PSCs to improve patient care and guide future innovation.

It is well known, and thus, not unexpected, that PSCs may develop quickly following ostomy creation surgery and that PSCs are more likely when leakage is present. The results of this ileostomy cohort sub-analysis highlight the importance of decreasing the risk of leakage, especially in harder-to-manage ostomy patients, such as people with a new ileostomy. It should be noted that the prevalence and impact of leakage in the acute care setting remain largely uncharacterized for institutions with shorter LOS or practices that discharge patients who continue to leak. Understanding the risk of leakage and its association with PSCs will be important to assess, along with ER visits or hospital readmission secondary to leakage or PSCs. Additionally, through reducing the risk of leakage in patients with a new ileostomy in the acute care setting, it is reasonable to anticipate that post-discharge patients’ confidence and quality of life will be higher, while the ostomy product consumption will decrease along with healthcare utilization necessary to manage leakage and PSC events.

### Strengths and Limitations

As described in the earlier publication, this study was retrospective in nature, and no prospective direct comparison of barrier pouching system types was conducted against ostomy types. Despite this, the retrospective design yields strength in supplying real-world evidence of barrier pouching system performance and the prevalence of leakage and PSCs in the acute care setting immediately following ostomy creation surgery. The study design also affords clinical insights into real-world patient demographics and surgical characteristics, which may affect the risk of leakage. Further, the robust WOC nurse practice at the study institution included ostomy education, care documentation, and staff training standardized between the two investigative sites, and barrier pouching system observations were only performed by a WOC nurse. These strengths allowed for a robust characterization of new ileostomy patients in the acute care setting and will enable hypothesis generation for future research on patients at higher risk of leakage and development of PSCs.

As reported in the earlier publication, data collection was performed by multiple WOC nurses, introducing the potential for reduced inter-rater reliability. To mitigate this concern, the investigators developed a standardized data collection tool that primarily utilized forced-response options to limit subjectivity. Comprehensive training sessions were conducted for all WOC nurse investigators, covering tool usage and standardized definitions for each field and term. In addition, quality assurance checks were implemented to further support consistency and enhance inter-rater reliability. While standardized education and care documentation is a strength of this study, the direct impact and relevance of these analyses are unknown as the nation-wide institutional standards of ostomy care vary greatly. Although WOC nurse expertise allows for specialization in ostomy care, the influence of this specialization on the prevalence of leakage and PSCs is also unknown as nurses who have not undergone WOC certification but regularly care for ostomy patients may still gain significant on-the-job experience in ostomy management. Lastly, despite a pre-determined definition of leakage, recording of leakage was binary as the severity of leakage (degree to which effluent extended under or beyond the barrier) was not consistently captured in the patient medical records, and therefore was not analyzed. The severity of leakage, and the correlation of degrees of leakage, to the onset of PSCs would be valuable to understand in future studies.

## 5. Conclusions

This sub-analysis demonstrates that ETB use is associated with a statistically significant 53.2% reduction in leakage risk among individuals with an ileostomy. The odds of leakage were 2.45 times higher with CIB compared to ETB. This difference is clinically meaningful, as ileostomy patients often face greater challenges with barrier adherence due to liquid and caustic effluent increasing the risk of PSCs. Evidence-based barrier selection can help clinicians minimize leakage, enhance patient confidence in ostomy management, improve health-related quality of life, reduce ostomy product consumption, and unnecessary healthcare resource utilization.

## Figures and Tables

**Table 1:** Demographic and surgical characteristics.

Patient Demographics	Total Study Population (N = 214)	Ileostomy Patients (N = 98)
Mean age years (SD) *	63.9 (13)	59.6 (14)
**Mean BMI kg/m^2^ (SD)**	27.9 (7)	27.9 (7)
**Gender**		
Male	106 (49.5%)	52 (53.1%)
Female	107 (50%)	45 (45.9%)
Transgender	1 (0.5%)	1 (1.0%)
**Race**		
African American	27 (13%)	11 (11.2%)
Asian	2 (1%)	2 (2.0%)
Non-specified	7 (3%)	2 (2.0%)
White	178 (83%)	83 (84.7%)
**Steroid use**		
No	179 (84%)	79 (80.6%)
Yes	36 (16%)	19 (19.4%)
**Surgical characteristics**		
**Reason for surgery**		
Chronic	108 (50.5%)	53 (54.1%)
Acute	106 (49.5%)	45 (45.9%)
Type of surgical approach *		
Laparoscopic	32 (15%)	3 (3.1%)
Open	176 (82.2%)	86 (87.8%)
Robotic	6 (3%)	9 (9.2%)
**Elective or emergent surgery**		
Elective	163 (76%)	53 (54.1%)
Emergent *	51 (24%)	45 (45.9%)
Surgery duration (min) *	252	277
End or loop *		
End	143 (66.8%)	50 (51%)
Loop	71 (33%)	48 (49%)
**Height of stoma os**		
Flush	23 (11%)	10 (10.2%)
Not described	1 (<1%)	0 (0%0
Protruding	183 (85%)	84 (85.7%)
Retracted	8 (3%)	4 (4.1%)
Effluent at first pouch change *		
Solid	5 (2.3%)	0 (0%)
Semi-liquid	49 (22.9%)	8 (8.2%)
Liquid	121 (56.5%)	87 (88.8%)
High output	1 (0.05%)	0 (0%)
Urine	33 (15.4%)	N/A
No output	5 (2.3%)	3 (3.1%)

* denotes statistical significance between the total study population and the ileostomy cohort, *p* < 0.05.

**Table 2:** Risk of leakage in the total study population and in ETB and CIB sub-groups.

Barrier Pouching System	Risk of Leakage— Total Study Population	Risk of Leakage— Ileostomy Patients
All barrier types	19%	18%
Ceramide-infused tape-border barrier (CIB) *	26%	29%
Elastic tapeless border barrier (ETB) *	15%	14%

* denotes statistical significance between CIB and ETB, *p* < 0.05.

## Data Availability

The original data presented in the study are openly available in the Journal of Wound Ostomy Continence Nursing at DOI: 10.1097/WON.0000000000001124.

## Public Involvement Statement

No public involvement in any aspect of this research.

## Guidelines and Standards Statement

This manuscript was drafted against The Strengthening the Reporting of Observational Studies in Epidemiology (STROBE) Statement: guidelines for reporting observational studies [19].

## Abbreviations

The following abbreviations are used in this manuscript:

CD	Crohn’s Disease
CIB	Ceramide-infused border barrier
ER	Emergency room
ETB	Elastic tapeless-border barrier
PSC	Peristomal skin complication
UPMC	University of Pittsburgh Medical Center
WOC	Wound, ostomy, continence
